# Adolescent self‐harm prevention and intervention in secondary schools: a survey of staff in England and Wales

**DOI:** 10.1111/camh.12308

**Published:** 2018-12-05

**Authors:** Rhiannon Evans, Rachel Parker, Abigail Emma Russell, Frances Mathews, Tamsin Ford, Gillian Hewitt, Jonathan Scourfield, Astrid Janssens

**Affiliations:** ^1^ DECIPHer School of Social Sciences Cardiff University Cardiff UK; ^2^ University of Exeter Medical School University of Exeter Exeter UK; ^3^ CASCADE School of Social Sciences Cardiff University Cardiff UK

**Keywords:** Adolescence, self‐harm, self‐injury, school, intervention

## Abstract

**Background:**

Adolescent self‐harm is a major public health concern. To date there is a limited evidence‐base for prevention or intervention, particularly within the school setting. To develop effective approaches, it is important to first understand the school context, including existing provision, barriers to implementation, and the acceptability of different approaches.

**Methods:**

A convenience sample of 222 secondary schools in England and Wales were invited to participate in a survey, with a 68.9% (*n* = 153) response rate. One member of staff completed the survey on behalf of each school. Participants responded to questions on the existing provision of adolescent self‐harm prevention and intervention, barriers to delivery, and future needs.

**Results:**

Adolescent self‐harm is an important concern for senior management and teachers. However, emotional health and well‐being is the primary health priority for schools. Health services, such as Child and Adolescent Mental Health Services, and on‐site counselling are the main approaches schools currently use to address adolescent self‐harm, with counselling cited as the most useful provision. Fifty‐two per cent of schools have received some staff training on adolescent self‐harm, although only 22% rated the adequacy of this training as high. Where schools do not have existing provision, respondents stated that they would like staff training, specialist student training, external speakers, posters and assemblies, although the latter four options were infrequently ranked as the most useful approaches. Key barriers to addressing adolescent self‐harm were: lack of time in the curriculum; lack of resources; lack of staff training and time; and fear of encouraging self‐harm amongst adolescents.

**Conclusions:**

Adolescent self‐harm is a priority for schools. Intervention might focus on increasing the availability of training to teaching staff.

**Key Practitioner Message**

- Self‐harm is a major public health concern amongst adolescents. Schools are key sites for prevention and intervention.
- Emotional health and wellbeing is the primary health priority for schools, although self‐harm is also a concern.
- Counselling is seen as the most useful school‐based provision to respond to adolescent self‐harm.
- Only 52% of schools have received staff training on self‐harm, with 22% of schools rating the adequacy of training as high.
- Key barriers to schools addressing adolescent self‐harm are: lack of time; lack of resources; lack of staff training and time; and fear of encouraging adolescents.

## Introduction

Self‐harm amongst adolescents is a major public health concern (Hawton, Saunders, & O'Connor, 2012). It may be defined as any act of self‐poising or self‐injury carried out by an individual irrespective of motivation (NICE, 2013). The median age of onset of self‐harm is reported to be 13 years (Morey, Mellon, Dailami, Verne, & Tapp, 2017), with the highest prevalence among girls (Kidger, Heron, Lewis, Evans, & Gunnell, 2012; Morey et al., 2017; Morgan et al., 2017). Community samples report that between 12.1% and 18.8% of adolescents have self‐harmed (Doyle, Treacy, & Sheridan, 2015; Kidger et al., 2012; Morey et al., 2017; Muehlenkamp, Claes, Havertape, & Plener, 2012). Incidence rates have risen in recent years, with a 68% increase amongst girls aged 13–16 years, from 45.9 per 10000 in 2011 to 77.0 per 10000 in 2014 (Morgan et al., 2017). Effective prevention and intervention for this population is important, as beyond the immediate risk of physical injury, self‐harm is a risk factor for unnatural death. Children and young people who have self‐harmed are more than 17 times as likely to die by suicide than those without a history of self‐harm (Morgan et al., 2017). Suicidal self‐harming behaviour is further associated with poorer educational attainment at age 16 years old and not being in education, employment, or training at 19 years old (Mars et al., 2014). However, those in full time education have been reported to be more likely to self‐harm, primarily to cope with anxiety (Young, van Beinum, Sweeting, & West, 2007).

Despite a proliferation in the number and range of interventions intended to address adolescent self‐harm, there is a limited number of effective approaches as established via a robust research design. The effectiveness of some therapeutic approaches has been reported (Hawton et al., 2015; Ougrin, Tranah, Stahl, Moran, & Asarnow, 2015), but further evaluation across a wider range of interventions is required, particularly within educational settings (Lake & Gould, 2011; Robinson, Calear, & Bailey, 2018; Robinson et al., 2013).Where school‐based approaches have developed, they may be categorised according to prevention or intervention (Robinson et al., 2013). Prevention primarily focuses on universal or indicated approaches that address education and knowledge or increase the identification of at‐risk individuals through screening. Meanwhile intervention largely refers to indicated approaches that assess and treat those where self‐harm has already been disclosed. A recent review by Robinson et al. (2018) identified only seven RCTs of school‐based interventions, three of which were universal and four of which were indicated. Five trials reported effectiveness, including the SEYLE study that found positive effects for the universal educational approach Youth Aware of Mental Health (Wasserman et al., 2015). Gatekeeper training has also demonstrated some effectiveness within this setting, for example the Signs of Suicide Prevention Programme (Aseltine, James, Schilling, & Glanovsky, 2007; Schilling, Aseltine, & James, 2016). The SEYLE study found no impact on training teachers as gatekeepers though, arguably due to their own poor wellbeing preventing them from being able to fully support students (Wasserman et al., 2015). Guidelines for school‐based management have also been issued, which include identification of at risk students, development of an initial response protocol, assessment of injury, and management of contagion and online activity (De Riggi, Moumne, Heath, & Lewis, 2017; Hasking et al., 2016). However, further evaluation of such recommendations needs to be undertaken.

To develop effective school‐based prevention and intervention, it is first important to understand current practices. This is because, in alignment with the complex systems perspective, intervention can be seen as an attempt to disrupt existing system dynamics, where entrenched structures and resources may work to support or reject the introduction of a new approach (Fletcher et al., 2016; Keshavarz, Nutbeam, Rowling, & Khavarpour, 2010; Moore & Evans, 2017). A recent systematic review and meta‐ethnography of international qualitative evidence on the role of schools in adolescent self‐harm and suicide found that it is often not prioritised (Evans & Hurrell, 2016). There is a culture of fear amongst staff (Best, 2006; Dowling & Doyle, 2017), with many school professionals feeling ill equipped to manage behaviours (Berger, Hasking, & Reupert, 2014a, 2014b; De Riggi et al., 2017). This often leads to the escalation of incidents through the hierarchical school structure in the effort to locate ‘expertise’, which often comes from an external source (Berger et al., 2014b; Best, 2006; McAndrew & Warne, 2014). If these management strategies are entrenched and recognised, they might prevent the introduction of school‐based interventions, impinge upon the activation of an intervention's mechanisms of change as intended, or influence what new practices might be deemed acceptable and feasible. To date there remains limited understanding of existing self‐harm provision or needs across secondary schools in the UK.

In this study we report survey data from a study of secondary school staff in England and Wales. The study addressed two primary research questions:

1. How do secondary schools in England and Wales currently prevent or intervene with adolescent self‐harm?
2. What prevention or intervention needs do secondary schools in England and Wales have in regard to adolescent self‐harm?

For the purpose of this study, we understand self‐harm as a broader category than self‐injury, as it includes both the infliction of damage to the external surface of the body and self‐poisoning (NICE, 2013). In accordance with the UK tradition, we did not differentiate self‐harm with or without an associated suicidal intent, as they are arguably continuously rather than bi‐modally distributed (Kapur, Cooper, O'Connor, & Hawton, 2013). As such, self‐harm could include non‐suicidal self‐injury, suicide attempts, self‐harm with an undetermined intent, or self‐harm with ambivalence.

## Methods

The study comprised a cross‐sectional survey with a convenience sample of secondary school staff in England and Wales. Data were collected between January and September 2016. Cardiff University's School of Social Sciences Research Ethics Committee provided ethical approval for the study (SREC/1849).

### Sample

Sampling and recruitment processes differed between England and Wales. In England, the sample comprised all state‐funded secondary schools in the South West counties of Devon and Somerset. In Wales, the sample comprised all state‐funded secondary schools enlisted in the School Health Research Network (SHRN), which is a research, policy, and practice infrastructure intended to improve the health and well‐being of young people. A total of 222 schools were eligible for participation, with 100 being located in South West England and 112 in Wales. Given the convenience sampling strategy, comparison to national data was used to examine representative of the study sample (see Table 1). The study response rate was 68.9% (*n* = 153), with a response rate of 59% (*n* = 59) in South West England and 83.9% (*n* = 94) in Wales.

**Table 1:** Comparison of sample and all schools in England and Wales (2014–2015 data)

	All schools in England (*n* = 4148)	South‐West England Sample (*n* = 100)	Responders South‐West England Sample (*n* = 59)	Non‐responders South‐West England Sample (*n* = 41)	All schools in Wales (*n* = 212)	Wales sample (*n* = 112)	Responders Wales Sample (*n* = 94)	Non‐responders Wales Sample (*n* = 18)
Mean disadvantaged students (%)^a^	28% (*SD* = 17, range = 2%–97%)	12% (*SD* = 6, range = 2%–33%)	12% (*SD* = 6, range = 2%–33%)	11% (*SD* = 5, range = 3.4%–27.6%)	17% (*SD* = 9, range = 4%–48%)	17% (*SD* = 10, range = 4%–48%)	17% (*SD* = 10, range = 4%–48%)	16% (*SD* = 10, range = 4%–43%)
Mean key stage 4 core subject indicator (%)^b^	59% (*SD* = 19, range = 2%–100%)	58% (*SD* = 17, range = 24%–100%)	59% (*SD* = 18, range = 24%–100%)	58% (*SD* 16, range = 31%–100%)	59% (*SD* = 12, range = 15%–89%)	59% (*SD* = 12, range = 24%–89%)	59% (*SD* = 11, range = 25%–87%)	60% (*SD* = 14, range 24%–89%)
Mean school size (*n*)^c^	863 (*SD* = 454, range = 11–3857)	924 (*SD* = 444, range = 77–2479)	936 (*SD* = 459, range = 77–2479)	904 (*SD* = 422, range = 105–2382	868 (*SD* = 372, range = 210–2202)	760 (*SD* = 273, range = 275–1503)	759 (*SD* = 269, range = 317–1503)	799 (*SD* = 332, range = 275–1265)

a England: Percentage of students eligible for free schools meals or looked after. Data are annual. Wales: Percentage of students eligible for free school meals. Data are 3‐year average.

b England: Percentage of students achieving 5+ A*‐C GCSEs or equivalents including A*‐C in both English and Mathematics. Wales: Percentage of students achieving 5+ A*‐C GCSEs or equivalents including A*‐C in both English or Welsh and Mathematics.

c Number of students enrolled at the school.

Schools nominated a member of staff with knowledge of existing school provision for adolescent self‐harm. In South West England, this staff member was identified through phone contact with the school. In Wales this individual was identified via the appointed SHRN contact. On completion of the survey respondents were asked to indicate their professional role. Cited roles were: assistant head teacher (60%); pastoral support (14%); safeguarding lead (7%); and other school professional (19%).

### Procedure

In South West England, respondents completed the survey online via SurveyMonkey (73%) or a paper version. In Wales, the survey was included as a supplement in the bi‐annual School Health Research Network (SHRN) school environment survey, which was completed in paper format. Participant consent was explained to the schools via an initial telephone conversation, where participants were informed that completion of the survey would be taken as informed consent. The survey had a set of instructions for completion, which included a statement on anonymity and confidentiality. Survey questions and items were chosen through consideration of the extant research literature on evaluated interventions and approaches schools currently use, stakeholder consultation, and discussion with members of the GW4 funded Children and Young People Suicide and Self‐harm Research Collaboration. GW4 is a consortium of the universities of Bath, Bristol, Cardiff, and Exeter aimed at enhancing research collaboration. In 2015 the GW4 Collaboration hosted a one‐day stakeholder consultation event to set the self‐harm research agenda and explore schools’ current provision and future needs. Many of the survey items were discussed by attending schools as routine practice or preferable/avoidable approaches. Questions explored: schools’ prioritisation of adolescent self‐harm; existing school provisions; staff training around adolescent self‐harm; the perceived adequacy of existing provisions, in addition to the adequacy of staff training; potential barriers to preventing or intervening; and future intervention needs (Appendix S1). The question on schools’ priorities was presented slightly differently between South West England and Wales due to the functionality of the different formats used, with the online survey used in England not being able to support the structure of the question which had already been included in the administered Welsh version. Respondents were provided with a suite of pre‐specified options to select from. Some questions included a free text option for respondents to expand on their selected answer. Free text responses were purposively sampled for inclusion in the results to represent schools across both countries and to reflect a range of perspectives. The survey was piloted with a subset of school staff to ensure relevance and readability.

### Analysis

Survey data were descriptively analysed with SPSS Version 23 (IBM, New York, NY). Data are summarised with *n* values and percentages.

## Results

### Schools’ health and well‐being priorities

Respondents reported on the health and well‐being priorities for their schools (Table 2). Schools in South West England were asked to indicate if the following areas of health and well‐being were a high priority: sex and relationships; suicide; smoking; emotional health and well‐being; alcohol; healthy eating; self‐harm; physical health; and substance use. Emotional health and well‐being were most frequently rated as a very high priority (61%), with self‐harm being rated as a very high priority by 37% of respondents.

**Table 2:** Schools health and well‐being priorities^a^

	How much of a priority are these health areas for your school? (South West England) (=59)					Rank the health priority areas of your school (Wales) (*n* = 94)								
	Very high priority (%)	High priority (%)	Moderate priority (%)	Low priority (%)	Very low priority (%)	1^st^ High priority (%)	2nd (%)	3rd (%)	4th (%)	5th (%)	6th (%)	7th (%)	8th (%)	9th Low priority (%)
Emotional health and well‐being	61	32	3	–	3	60	10	5	6	5	1	10	1	2
Sex and relationships	39	39	17	3	2	5	14	22	25	11	10	12	1	–
Self‐harm	37	36	20	3	3	5	20	17	10	7	2	10	27	2
Physical health	36	39	24	–	–	7	16	9	10	15	7	10	9	17
Suicide	32	27	24	14	3	13	10	9	8	4	4	2	10	40
Drugs	32	52	16	–	–	4	10	9	16	25	11	9	12	5
Alcohol	22	44	32	2	–	1	4	11	10	15	27	12	11	9
Healthy eating	20	46	32	–	2	2	11	12	9	7	22	11	13	12
Smoking	17	46	34	–	3	2	10	6	6	17	19	21	12	6

a Totals may not equal 100% due to rounding.

Welsh schools ranked the health priorities from one to nine. Emotional health and well‐being were endorsed as the highest priority, with 60% ranking it first. Self‐harm and suicide ratings had a different profile compared to the other health areas, as the distribution was bimodal. Forty two per cent of respondents rated self‐harm as one of the top three priorities, whilst 29% ranked it as the 8th or 9th priority.

### Existing provision for adolescent self‐harm prevention and intervention

Respondents reported existing adolescent self‐harm and prevention activities currently provided by the school (Table 3). Health services, such as Child and Adolescent Mental Health Services (CAMHS), were cited as one of the main provisions, being routinely accessed by 82% of schools. CAMHS is a local NHS service across England and Wales that assesses and treat individuals aged <18 years with emotional and behavioural difficulties. CAMHS teams are multi‐disciplinary, comprising, amongst others, psychologists, support workers, and social workers. Referral to CAMHS may be made by a guardian or professional, such as a GP or educational practitioner. Schools primarily make student referrals to offsite CAMHS services, but on occasion CAMHS may deliver some onsite training or awareness raising. Other approaches utilised by schools included on‐site counselling, school policies and procedures (e.g. safeguarding procedures) and drop in onsite health services that may discuss self‐harm with students. These health services are largely delivered by the school's pastoral team, occasionally through a designated student hub.

Respondents were asked to identify areas where they do not currently have provision, but would like to. These included specialist training to students around self‐harm (36%), posters on self‐harm (27%), outside speakers or organisations (25%), training for staff on student self‐harm (22%), and assemblies about adolescent self‐harm (21%).

**Table 3:** Existing adolescent self‐harm prevention and intervention provision^a^

	Routinely provided (%)	One off provision (%)	Not currently provided (%)	Not currently provided, but would like to provide (%)
Health services (e.g. CAMHS) (*n* = 153)	82	10	1	7
On‐site counselling (*n* = 151)	79	10	3	8
Drop‐in health services (*n* = 151)	75	13	3	9
School policies and procedures (*n* = 153)	75	11	2	12
PSHE (*n* = 145)	41	33	12	14
Training for staff (*n* = 152)	38	29	11	22
Posters (*n* = 150)	32	9	32	27
Assemblies (*n* = 150)	23	32	24	21
Outside speakers or organisations (*n* = 150)	15	34	25	25
Specialist training to students (*n* = 148)	7	22	34	36

a Totals may not equal 100% due to rounding.

Respondents were asked to indicate who within the schools took responsibility for the delivery of existing provisions around adolescent self‐harm. A high proportion of school‐based staff were involved: pastoral team (97%); senior management (86%); teaching support staff (79%); and teachers (74%). Respondents also reported the involvement of: school nurses (91%); school counsellors (92%); CAMHS (92%); allied health professionals (34%); and the voluntary sector (18%). Students were reported to be involved in supporting their peers in 45% of schools.

### Adequacy of existing student self‐harm prevention and intervention provision

Respondents were asked to rank the five most useful self‐harm prevention and intervention approaches (Table 4). Counsellors were ranked as the most useful approach, accounting for 25% of all provisions ranked first. This was followed by CAMHS (14%) and staff training on adolescent self‐harm (12%). Provisions that were not perceived as most useful were awareness raising training, whole school approaches, and posters.

**Table 4:** Adequacy of adolescent self‐harm prevention and intervention provision^a^

	1^st^ Most useful (%) (*n* = 129)	2nd Most useful (%) (*n* = 128)	3rd Most useful (%) (*n* = 128)	4th Most useful (%) (*n* = 125)	5th Most useful (%) (*n* = 124)
Counsellor	25	13	14	6	6
CAMHS	14	11	16	13	10
Staff training	12	10	13	17	15
School Policies and Procedures	9	17	14	7	10
PSHE	9	8	5	9	15
Student drop‐in	8	15	11	15	9
Student training	8	9	3	6	4
Outside speakers	3	7	11	6	8
One‐to‐one support	3	2	–	1	2
Assemblies	2	2	4	2	6
Student support programme	2	–	1	1	1
Awareness raising	1	2	2	1	–
Whole school approaches	1	1	1	2	–
Posters	1	–	–	5	6
Other	2	3	5	8	8

a Totals may not equal 100% due to rounding.

### Staff training on adolescent self‐harm prevention and intervention

Data were collected on the specific training that school staff members have received in regard to student self‐harm. Fifty‐two per cent of respondents reported that their school had received some staff training. Fifty‐eight per cent of schools in South West England (*n* = 34) had received staff training compared to 50% in Wales (*n* = 46). A number of participants did not know if the school had received training (7%). Fourty per cent of schools had not received any staff training.

For all schools that indicated receipt of staff training, 84% reported the training source. CAMHS were the most frequently cited trainer (31%). Twelve per cent reported in house training, 12% reported primary mental health team training, and 10% reported training from a charity.

### Adequacy of staff training on adolescent self‐harm prevention and intervention

Respondents rated the specific adequacy of current staff training around adolescent self‐harm. For all schools, 50% (*n* = 74) of individuals who responded to this question (*n* = 148) indicated that the adequacy of staff training is moderate. Meanwhile 22% endorsed the current adequacy of provision as being very high or high (*n* = 33). Twenty‐four per cent of respondents stated that training adequacy was low or very low, with this being higher in Wales (28%, *n* = 25) than England (19%, *n* = 11). Five per cent of schools, all of which were in England, stated that no training was provided.

Respondents were asked to expand on the reasons for their rating of training adequacy. Fifty‐one respondents in Wales and 52 in England provided free‐text comments. Explanations of high adequacy focused on schools’ prioritisation of self‐harm, with responses stating: ‘[self‐harm is] *very much at the forefront and* [the school] *have a clear strategy to address the issue’*. Explanations of moderate ratings often centred on schools’ reactive approach: *‘we respond to need and could be more proactive’* and *‘strong individual support, but few proactive strategies’*. Examples of explanations for low adequacy ratings included:

Little support available from school nurse/health service since we lost our allocated school nurse. It is now a team, which we rarely see and they do not engage with our students. Rarely get support from CAMHS unless serious case – need advice on prevention.

Staff are not sufficiently trained to deal with self‐harm. A school's core business is to educate young people. We refer to specialists for example, CAMHS/counsellor to deal with specific cases.

### Barriers to school‐based self‐harm prevention and intervention

Respondents indicated the key barriers to adolescent self‐harm prevention and intervention within schools (Table 5). Frequent responses were lack of time in the curriculum (47%), inadequate training or time for school staff (42%), and limited resources (38%). Thirty six per cent of respondents stated that fear of encouraging students was a barrier. There were some differences across sites in terms of staff training, with 32% of respondents in South West England (*n* = 19) and 49% (*n* = 45) of respondents in Wales identifying lack of staff training as a major issue.

**Table 5:** Barriers to adolescent self‐harm prevention and intervention^a^

	Major barrier (%)	Minor barrier (%)	Not a barrier (%)
Lack of time in curriculum to deliver activities (*n* = 150)	47	31	21
Inadequate training for school staff (*n* = 151)	42	39	19
Lack of available resources (*n* = 150)	38	36	26
Fear of encouraging students (*n* = 150)	36	44	20
Lack of staff time to deliver activities (*n* = 150)	36	37	27
Other health topics given higher priority (*n* = 150)	11	47	41
Students fail to engage with the topic (*n* = 149)	3	23	74
Not seen as problem by teachers (*n* = 150)	3	19	78
Not seen as a problem by senior management (*n* = 150)	3	9	88
School not an appropriate place (*n* = 150)	1	15	83

a Totals may not equal 100% due to rounding.

Attitudinal responses to self‐harm were least frequently endorsed as barriers. Self‐harm not being seen as a problem by senior management or teachers was not seen as a barrier. Equally, 74% stated that students’ failure to engage in the topic was not a barrier.

## Discussion

This study surveyed secondary schools in South West England and Wales to explore existing adolescent self‐harm prevention and intervention provision, barriers to delivery, and future needs. Through consideration of current practice, we can start to understand how the complex school system might respond to efforts to develop evidence‐based approaches that are acceptable and feasible within this context. This is important given the current lack of effective self‐harm interventions, or research‐informed recommendations within the UK.

Nominated school staff understood self‐harm to be a health priority and when considering barriers to intervention, schools not being an appropriate place was rarely cited as an issue. However, emotional health and well‐being was cited as the main priority across England and Wales. As part of their current approach, it appears that schools tend to focus on indicated intervention rather than prevention, primarily relying on escalating incidents of self‐harm to internal or external experts, notably Child and Adolescent Mental Health Services (CAMHS) and school counsellors. This aligns with documented practices in the international literature, where school staff tend to make referrals to mental health professionals such as psychologists (Berger et al., 2014a).

Schools cited counsellors and specialist health provisions as being most adequate in addressing adolescent self‐harm. Yet despite an extensive policy focus on supporting CAMHS and school‐based counselling across England and Wales (Department for Education, 2016; Public Health England, 2015; Welsh Government, 2012), research has identified significant barriers to accessing this support. This includes the high diagnostic thresholds for access to CAMHS teams or the limited capacity of the service (Rice, Eyre, Riglin, & Potter, 2017; Sharpe et al., 2016). Similar problems have been identified internationally, with concerns around a lack of federal investment in schools’ access to mental health professionals (Maag & Katsiyannis, 2010). As schools are heavily reliant on these services, it is important that they are sufficiently funded. Recent UK government action has sought to improve relationships between CAMHS and schools, notably the Welsh Government's investment of £1.4 m in specialist CAMHS practitioners to provide specialist liaison, consultancy and advice to teaching staff (Welsh Government, 2017). The effects of these ongoing commitments need to be established. The training available to specialist mental health professionals to support schools in intervening with incidents of adolescent self‐harm also needs consideration, as many report feeling ill‐equipped (Best, 2006).

Limited access to mental health professionals is particularly problematic due to the variable provision of staff training. Only just over half of respondents stated that staff had received dedicated training on adolescent self‐harm, despite being indicated as one of the most useful provisions. Meanwhile a lack of training was cited as one of the most significant barriers to effectively addressing the issue. Similar findings have been reported elsewhere (Berger et al., 2014a, 2014b; Dowling & Doyle, 2017), with a survey of teachers in Australia finding that 41% require improved staff education and school policy frameworks (Berger et al., 2014a).

Schools indicated areas where they would like to offer provision in the future. These included specialist training to students, posters, external speakers and assemblies. However, whilst these approaches were indicated as acceptable to schools, they were infrequently cited as the most adequate provision for addressing adolescent self‐harm. This result might be due to the structuring of the question, where schools were asked to suggest approaches that were not provided and they would like to provide in future; these options were not frequently cited as existing practice and may be seen as *additional* activities that may be undertaken.

In considering the potential introduction of universal approaches to self‐harm, caution must be exercised due to the possible risks associated with them. Indeed, one of the key barriers mentioned by schools was a fear that addressing self‐harm directly might encourage students to engage in such practices. To date there is limited evidence around the potential iatrogenic effects of self‐harm prevention or intervention amongst adolescents within the school context, although they have rarely been reported within evaluation studies (Robinson et al., 2018). Where adverse effects have been assessed across a range of suicide‐related outcomes, it has been found that asking about ideation does not increase the risk (Mathias et al., 2012; Robinson et al., 2018), although a number of awareness raising sessions around suicide have been shown to have a detrimental impact upon attitudes or cause distress amongst those with a history of attempt (Shaffer, Garland, Vieland, Underwood, & Busner, 1991; Shaffer et al., 1990). Schools then need to be supported in understanding how to talk to students about self‐harm, and where universal approaches such as assemblies or posters are to be used, they require information on the nature and depth of information to be shared (Whitlock & Rodham, 2013).

Staff training was also indicated as a provision that schools want more of in the future. However, whilst the provision of training may be a positive step, we need to be aware of the quality of training that schools receive, and its underpinning evidence base. Respondents were asked to rate the quality of staff training they had received to date and only 22% considered the quality to be very high or highly adequate. There is a significant literature on the impact of gatekeeper training, which provides a useful direction for the training that might be delivered to staff (Isaac et al., 2009). Such training has been shown to increase knowledge, skills, and attitudes around suicide. Continuous work is required to evaluate these interventions, including more robust evaluation of the Signs of Self‐Injury (Muehlenkamp, Walsh, & McDade, 2010). These is also a need to consider the implementation of such interventions within the UK educational context, especially given reported issues around the referral patterns for gatekeepers elsewhere (Isaac et al., 2009). Further work may also attend to the well‐being of teachers in their role supporting students, as poor well‐being has been theorised to compromise the potential effectiveness of gatekeeper type approaches (Wasserman et al., 2015). At the system level, schools may also be supported in developing research informed guidelines similar to those that have been issued in the USA and Canada (De Riggi et al., 2017; Hasking et al., 2016).

Whilst the data from this study may serve as a useful departure point for researchers, practitioners, and policy‐makers to better support schools in addressing adolescent self‐harm, it is important to recognise the enduring structural constraints that schools are continually subjected to. The main barriers to self‐harm prevention and intervention centred on the lack of resources and lack of time, particularly time within the curriculum, which have also been identified within the international literature (Berger et al., 2014a). In order to mitigate such barriers, structural reform may be required, which involves a keener prioritisation of health and well‐being as part of the core business of schools. In the UK for example, the Welsh Government is undertaking a significant school curriculum review that focuses on the holistic development of children, foregrounding the importance of opportunities to build well‐being through developing confidence, resilience, and empathy (Welsh Government, 2015). Meanwhile, in England Personal, Social, Health and Economic (PSHE) is on course to becoming mandatory, opening up opportunities for more comprehensive health‐based activities. Such approaches may encourage sufficient dedication of resources to health, although the consequences of such reforms are yet to be realised and will require future evaluation.

### Implications

Drawing the findings together, it is evident that the study has a number of key implications for policy and practice. First, there needs to be a consistent effort to provide high quality training support to school staff so that they are more confident to respond to disclosures of self‐harm. Second, whilst clinical developments within the field of self‐harm intervention are notable (De Riggi et al., 2017), there remains limited capacity amongst mental health services to meet the needs of schools requiring external, specialist support (Sharpe et al., 2016). Investment is required to improve access. Third, in order to support school‐level intervention, a coordinated policy approach needs to be adopted in order to ensure that sufficient resources are dedicated to adolescent mental health and well‐being and it is prioritised within this setting.

### Strengths and limitations

The survey collated data from a large sample, with comprehensive coverage across South‐West England and Wales. The convenience sample presented some differences compared to the national average on student deprivation, academic attainment, and school size. Representativeness may be limited in Wales as schools were self‐selected members of the School Health Research Network and likely had a preexisting interest in research. Generalisability may be limited in England as data were collected from one geographical region. Further research might replicate this study with a larger representative sample.

For the majority of questions, the same format was employed across South West England and Wales, but due to differences in the functionality of the formats used to undertake the survey (e.g. online vs paper format) the structure of the question on schools’ health priorities had to be adapted across the two sites. As a result, this data is not comparable and should be interpreted with caution.

Comparability of survey data across respondents was also limited by the non‐standardisation of responding professionals. Schools were asked to identify the member of staff with the most comprehensive knowledge of self‐harm prevention and intervention provision, and as a result there was variation in reported roles. As such understandings of self‐harm practices and awareness of provision might vary. Equally, in asking schools to identify the respondent with most knowledge of self‐harm provision, the extent to which it is seen to be a priority or the extensiveness of existing prevention and intervention activity may be overstated. However, pragmatically it was important to identify schools’ current practices, and these individuals were best placed to provide this information. The fact that this professional role varied between schools is also useful in understanding where knowledge and expertise is located within the school setting, which may support future intervention development work.

Finally, the survey asked respondents to consider both prevention and intervention needs within the same questions. For example, for the question on barriers and facilitators to delivery, respondents were providing overall assessments for prevention and intervention, when different barriers to universal prevention or indicated intervention may be encountered. Moreover, a common limitation with surveys is that participants may have interpreted the survey items differently.

## Conclusion

Although emotional health and well‐being is the primary health concern for schools, self‐harm is a priority. Schools currently rely upon professional mental health services such as CAMHS and onsite counselling for intervention provision, with the latter being cited as the most useful approach. Schools find staff training to be useful, but only just over half of schools have received such training, and it is not generally considered to be highly adequate. As almost three quarters of teachers are reported to be involved in addressing adolescent self‐harm, staff training should be considered a priority. Structural barriers to prevention persist, including lack of time and resources, and longer‐term school reform that prioritises health and well‐being alongside education may be required. Further research needs to be undertaken to explore other cited barriers to prevention and intervention, specifically fears about encouraging students to engage in such practices.

## Ethical information

Data were collected between January and September 2016. Cardiff University's School of Social Sciences Research Ethics Committee provided ethical approval for the study (SREC/1849). Participant consent was explained to the schools via an initial telephone conversation, where participants were informed that completion of the survey would be taken as informed consent.

## Correspondence

Rhiannon Evans, DECIPHer, School of Social Sciences, Cardiff University, 1‐3 Museum Place, Cardiff CF10 3BD, UK; Email: EvansRE8@cardiff.ac.uk

## Supporting information


**Appendix S1.** School environment questionnaire 2015–16.
