# Supplementary material for: Adolescent self‐harm prevention and intervention in secondary schools: a survey of staff in England and Wales
Source: Child Adolesc Ment Health. 2018 Dec 5;24(3):230–8. doi: 10.1111/camh.12308 (PMC6767699; doi:10.1111/camh.12308)
Supplement: Supplementary file 1 — Appendix S1. School environment questionnaire 2015–16. [file CAMH-24-230-s001.pdf]

Y RHWYDWAITH YMCHWIL
IECHYD MEWN YSGOLION

SCHOOL HEALTH
RESEARCH NETWORK

SCHOOL HEALTH RESEARCH NETWORK
SCHOOL ENVIRONMENT QUESTIONNAIRE 2015-16

Information and Guidance for School Survey Lead

Purpose of the School Environment Questionnaire

The information you provide in the questionnaire will be used for two main purposes:

1. To support research into different aspects of the school environment and how they influence student health and wellbeing.

2. To provide monitoring data to the Welsh Network of Healthy School Schemes (WNHSS).

The answers you provide to the core section of the questionnaire will be shared with the WNHSS in a non-anonymised form to support planning, monitoring and development of local Healthy School Schemes and the national network. It is important that the School Health Research Network and WNHSS work together to effectively and efficiently advance school health and wellbeing in Wales. Sharing the school environment questionnaire data in this way reduces duplication of effort and burden on schools as it means the WNHSS will not have to undertake further data collection to meet their information needs. Information you provide about your school will be held in strictest confidence by WNHSS and no data that could be linked to an individual school will ever be published.

Format of the Questionnaire

There are two parts to the questionnaire: the core (questions 1 to 60) and the supplement (questions 61 to 70). Each time the questionnaire is completed (every two years), the core will remain largely unchanged and the supplement will focus on a particular issue in depth.

This year, the supplement focuses on self-harm, reflecting the concerns schools have raised. The data will be used to inform the next steps in a research project that aims to develop a school-based intervention on self-harm. It will not form part of the monitoring data shared with WNHSS.

Completing the Questionnaire

There is one questionnaire per school and it should be completed by a member of the senior management team. Other staff may need to be consulted in order to answer all the questions.

Please answer all the questions by placing a tick in the appropriate box and providing a written answer where requested. Depending on your answers, some questions can be skipped; instructions alongside the questions will guide you through this.

If your school has students in years 12 and 13, please include your policies and practices pertaining to all year groups unless the question specifies years 7 to 11 only. If you don't have a 6th form, leave questions pertaining to these year groups blank.

If you have any questions, please get in touch with Gillian or Joan.
Email: shrn@cardiff.ac.uk   Tel: 029 2087 9609.

When you are sure you have completed the questionnaire, please return it the School Health Research Network using the freepost envelope provided. If you lose your envelope, please contact us for a replacement or post your questionnaire to us at:

DECIPHer, Cardiff University, 1-3 Museum Place, Cardiff CF10 3BD

Please return your questionnaire by **Thursday 24th March**.

Please answer as much of the questionnaire as you can *- the data you provide is very valuable and will further our understanding of how schools can protect and improve young people's health and wellbeing.*

Thank you.

GENERAL QUESTIONS ABOUT YOU

Q1 Which of the following best describes your current professional role within the senior management team or senior leadership team?

PLEASE TICK ONE BOX ONLY

- ☐ Headteacher
- ☐ Acting headteacher
- ☐ Deputy headteacher
- ☐ Assistant headteacher
- ☐ Other **(PLEASE TICK AND WRITE IN BELOW)**

✍

Q2 In the 2013/14 and 2014/15 school years, in which of the following areas did the senior management team focus their efforts to make improvements.

TICK UP TO *FOUR* PRIORITY AREAS ONLY

- ☐ Staff health and wellbeing
- ☐ Staff relationships with students
- ☐ Student physical health (e.g. smoking, obesity)
- ☐ Student mental and emotional health
- ☐ Student educational attainment
- ☐ Estyn report and categorisation
- ☐ The physical condition of the school buildings and grounds
- ☐ The provision of extra-curricular activities
- ☐ Your school's relationships with parents
- ☐ Your school's relationships with the local community

GENERAL QUESTIONS ABOUT YOUR SCHOOL

Q3a Does your school have a **single** strategic lead for student health and wellbeing?

PLEASE TICK ONE BOX ONLY

- ☐ Yes, one individual leads on health and wellbeing (**GO TO Q3b)**
- ☐ No, one individual leads on health and one on wellbeing **(GO TO Q3b)**
- ☐ No, different individuals lead on different health and wellbeing areas and/or different phases (**GO TO Q3b)**
- ☐ No, no one leads (**GO TO Q4a)**

Q3b If you have either a single lead or multiple leads for school health and wellbeing, what is the professional role of the person or persons who lead?

PLEASE TICK ALL THAT APPLY

- ☐ Headteacher / Acting headteacher
- ☐ Deputy headteacher
- ☐ Assistant headteacher
- ☐ Head of phase
- ☐ Head of year
- ☐ Head of department
- ☐ Subject teacher
- ☐ School nurse
- ☐ Other (**PLEASE TICK AND WRITE IN BELOW**)

✍ ______________________________

Q4a Is your school currently participating in the local healthy school scheme as part of the Welsh Network of Healthy School Schemes (WNHSS)?
PLEASE TICK ONE BOX ONLY

☐ Yes **(GO TO Q4b, c and d)**

☐ No **(GO TO Q5a)**

Q4b **IF YES AT Q4a,** in which year did your school join the scheme?
PLEASE WRITE IN THE BOXES BELOW e.g. 2012

Q4c **IF YES AT Q4a**, what level has your school attained?
PLEASE TICK ONE BOX ONLY

☐ We are working towards phase 1

☐ We have achieved phase 1

☐ We have achieved phase 2

☐ We have achieved phase 3

☐ We have achieved phase 4

☐ We have achieved phase 5

☐ We have achieved phase 6 (locally accredited)

☐ We have achieved the National Quality Award

Q4d **IF YES AT Q4a,** in which year did your school achieve its current level?
PLEASE WRITE IN THE BOXES BELOW e.g. 2012
LEAVE BLANK IF YOU ARE WORKING TOWARDS PHASE 1

Q5a Does your school have a written school health and wellbeing action plan or school health and wellbeing targets?
PLEASE TICK ONE BOX ONLY

☐ Yes **(GO TO Q5b and c)**

☐ No **(GO TO Q6a)**

Q5b **IF YES AT Q5a,** are your action plan or targets included in your current School Development Plan?
PLEASE TICK ONE BOX ONLY

☐ Yes

☐ No

Q5c **IF YES AT Q5a,** does the Senior Management / Leadership Team formally assess progress against the action plan or targets?
PLEASE TICK ONE BOX ONLY

☐ Yes, at least annually

☐ Yes, less than annually

☐ No

Q6a If your school uses data (e.g. student surveys) to update its policies and practices on creating a healthy school, where do the data come from?
PLEASE TICK ALL THAT APPLY

Our data come from:

- ☐ Our own student surveys
- ☐ Estyn student survey
- ☐ Our own staff surveys
- ☐ Our own parent surveys
- ☐ Local authority data
- ☐ Routinely collected school data
- ☐ School Health Research Network Student Health & Wellbeing Report
- ☐ Other (**PLEASE TICK AND WRITE IN BELOW**)
- ☐ Our school does not use data for this purpose **(GO TO Q7a)**

Q6b **If your school uses data,** who uses it?
PLEASE TICK ALL THAT APPLY

Our data is used by:

- ☐ Senior management team
- ☐ School council
- ☐ School governors
- ☐ Healthy schools coordinator
- ☐ Other (**PLEASE TICK AND WRITE IN BELOW**)

Q6c **If your school uses data,** how do you use the data?
PLEASE TICK ALL THAT APPLY

We use our data to:

- ☐ Identify need and set health and wellbeing priorities
- ☐ Measure the impact of our work on creating a healthy school
- ☐ Look at how health and wellbeing at our school compares with other local schools or schools within our family

Q7a Does your school have a School Council or similar student-led body?
PLEASE TICK ONE BOX ONLY

- [ ] Yes **(GO TO Q7b and c)**
- [ ] No **(GO TO Q8)**

Q7b **IF YES AT Q7a**, how many times a year does it meet?
PLEASE TICK ONE BOX ONLY

- [ ] Less than 6 times
- [ ] More than 6 times
- [ ] 6 times

Q7c **IF YES AT Q7a**, how are students identified for membership of the school council?
PLEASE TICK ALL THAT APPLY

- [ ] Members chosen by staff
- [ ] Self-nominated candidates voted for by students
- [ ] Staff-nominated candidates voted for by students
- [ ] Other
- [ ] Student-nominated candidates voted for by students

Q8a How long do students have for their lunch break at your school?
PLEASE TICK ONE BOX ONLY

- [ ] Less than 30 minutes
- [ ] 50 or 55 minutes
- [ ] 30 or 35 minutes
- [ ] 60 or more minutes
- [ ] 40 or 45 minutes

Q8b Have you considered changing the length of your lunch break in the last three years?
PLEASE TICK ALL THAT APPLY

- [ ] No
- [ ] Yes, and we reduced it
- [ ] Yes, we considered reducing it, but decided not to
- [ ] Yes, and we increased it
- [ ] Yes, we considered increasing it, but decided not to

Q9a Are any students in years 7 to 11 allowed off school premises during their lunch break?

PLEASE TICK ONE BOX ONLY

☐ Yes **(GO TO Q9b)**

☐ No **(GO TO Q10a)**

Q9b **IF YES AT Q9a,** which year groups are allowed off school premises?

TICK ONE BOX ONLY ON EACH LINE

	All students	Only students with parental consent	No students
Year 7	☐	☐	☐
Year 8	☐	☐	☐
Year 9	☐	☐	☐
Year 10	☐	☐	☐
Year 11	☐	☐	☐

SCHOOL HEALTH AND WELLBEING POLICY

Q10 Does your school have written policies that include the following health and wellbeing areas?

PLEASE TICK ONE BOX ON EACH ROW

	Yes	In development	No
Smoking and tobacco use	☐	☐	☐
Drugs	☐	☐	☐
Alcohol	☐	☐	☐
Healthy eating or Food & Fitness	☐	☐	☐
Mental health and wellbeing	☐	☐	☐
Suicide prevention and/or post-suicide care	☐	☐	☐
Violence against women and girls	☐	☐	☐

Q11 Were students involved in developing any of the policies listed below?
For each policy, please indicate whether and how students were involved.

	No student involve-ment	Consult-ations with school council	Consult-ations with other student voice groups	Wider consult-ations with students (e.g. surveys)	Suggestion boxes	Other	Don't have a policy
Smoking and tobacco use	☐	☐	☐	☐	☐	☐	☐
Drugs	☐	☐	☐	☐	☐	☐	☐
Alcohol	☐	☐	☐	☐	☐	☐	☐
Healthy eating or Food & Fitness	☐	☐	☐	☐	☐	☐	☐
Mental health and wellbeing	☐	☐	☐	☐	☐	☐	☐
Behaviour and discipline	☐	☐	☐	☐	☐	☐	☐
Bullying	☐	☐	☐	☐	☐	☐	☐
Suicide prevention and/or post-suicide care	☐	☐	☐	☐	☐	☐	☐
Sex and relationships	☐	☐	☐	☐	☐	☐	☐
Violence against women and girls	☐	☐	☐	☐	☐	☐	☐

Q12 Which of the following methods do you use to communicate your school's written health and wellbeing policies?

TICK ALL THAT APPLY

- ☐ School newsletter
- ☐ Verbal communication to staff, e.g. staff meetings
- ☐ Verbal communication to students, e.g. assembly
- ☐ Verbal communication to parents, e.g. parents' evening
- ☐ Publication online via school website
- ☐ Other electronic distribution, e.g. email to students
- ☐ Other written distribution, e.g. student handbook
- ☐ Posters / signs around school
- ☐ None of the above

Q13 How often are the following school health policies reviewed?
PLEASE TICK ONE BOX ON EACH ROW

	Don't have a policy	At least once a year	Less than once a year, but at least every 3 years	Less than once every 3 years	Never been reviewed	Policy less than 1 yr old
Smoking and tobacco use	☐	☐	☐	☐	☐	☐
Drugs	☐	☐	☐	☐	☐	☐
Alcohol	☐	☐	☐	☐	☐	☐
Healthy eating or Food & Fitness	☐	☐	☐	☐	☐	☐
Mental health and wellbeing	☐	☐	☐	☐	☐	☐
Behaviour and discipline	☐	☐	☐	☐	☐	☐
Bullying	☐	☐	☐	☐	☐	☐
Suicide prevention and/or post-suicide care	☐	☐	☐	☐	☐	☐
Sex and relationships	☐	☐	☐	☐	☐	☐
Violence against women and girls	☐	☐	☐	☐	☐	☐

PARENTAL INVOLVEMENT IN HEALTH AND WELLBEING IMPROVEMENT

Q14a What proportion of parents does your school ***attempt to involve*** in **decisions** regarding health and wellbeing improvement?

PLEASE TICK ONE BOX ONLY

- ☐ None
- ☐ A few
- ☐ About half
- ☐ Most
- ☐ All

Q14b What proportion of parents do you think ***are involved*** in **decisions** regarding health and wellbeing improvement?

PLEASE TICK ONE BOX ONLY

- ☐ None **(GO TO Q15)**
- ☐ A few
- ☐ About half
- ☐ Most
- ☐ All

Q14c In what areas are parents involved in decision making?

PLEASE TICK ALL THAT APPLY

- ☐ Identifying health priority areas
- ☐ Health education
- ☐ School health policy
- ☐ Other

Q14d In what ways are parents involved in health and wellbeing improvement decisions?

PLEASE TICK ALL THAT APPLY

- ☐ PTA meeting
- ☐ Surveys
- ☐ Information evenings for parents
- ☐ As parent governors
- ☐ One-to-one meetings with parents
- ☐ Pressure from parents (i.e. contact initiated by parents on health issues)
- ☐ Other

HEALTH AND WELLBEING EDUCATION WITHIN THE CURRICULUM

Q15 How regularly do students within each year group in your school receive PSE or PSE provision within the Welsh Baccalaureate?

PLEASE TICK ONE BOX FOR EACH YEAR GROUP IN YOUR SCHOOL

	At least weekly	Less than weekly, but at least monthly	Less than monthly
Year 7	☐	☐	☐
Year 8	☐	☐	☐
Year 9	☐	☐	☐
Year 10	☐	☐	☐
Year 11	☐	☐	☐
Year 12	☐	☐	☐
Year 13	☐	☐	☐

Q16 How long, on average, are PSE sessions (either as PSE lessons or within Welsh Baccalaureate) at your school?

Please note down the answer in minutes e.g. 045.

If you have a 2 week timetable, please average the allocation for 1 week.

*If you **only** deliver PSE through PSE days, leave blank.*

☐☐☐ minutes

*If you use a collapsed timetable to deliver all your PSE (i.e. PSE days) **OR** you run PSE days **in addition to** regular timetabled sessions, how many days are you holding this year per year group?*

Year 7	☐☐	days
Year 8	☐☐	days
Year 9	☐☐	days
Year 10	☐☐	days
Year 11	☐☐	days
Year 12	☐☐	days
Year 13	☐☐	days

Q17 For each year group, how much PE is timetabled weekly within the formal curriculum?

If you have a 2 week timetable, please average the allocation for 1 week. Please note down the answer in minutes e.g. 045.

Year 7 ☐☐☐ minutes

Year 8 ☐☐☐ minutes

Year 9 ☐☐☐ minutes

Year 10 ☐☐☐ minutes

Year 11 ☐☐☐ minutes

Q18 Which year groups receive education on healthy eating within the formal curriculum and where is it taught?

PLEASE TICK ALL THAT APPLY FOR EACH YEAR GROUP IN YOUR SCHOOL

	PSE or Welsh Baccalaureate	Science	Food technology	Other	Not taught to this year group
Year 7	☐	☐	☐	☐	☐
Year 8	☐	☐	☐	☐	☐
Year 9	☐	☐	☐	☐	☐
Year 10	☐	☐	☐	☐	☐
Year 11	☐	☐	☐	☐	☐
Year 12	☐	☐	☐	☐	☐
Year 13	☐	☐	☐	☐	☐

Q19 Which year groups receive education on physical activity (**<u>other than during PE</u>**) within the formal curriculum and where is it taught?

PLEASE TICK ALL THAT APPLY FOR EACH YEAR GROUP IN YOUR SCHOOL

	PSE or Welsh Baccalaureate	Science	Vocational courses	Other	Not taught to this year group
Year 7	☐	☐	☐	☐	☐
Year 8	☐	☐	☐	☐	☐
Year 9	☐	☐	☐	☐	☐
Year 10	☐	☐	☐	☐	☐
Year 11	☐	☐	☐	☐	☐
Year 12	☐	☐	☐	☐	☐
Year 13	☐	☐	☐	☐	☐

Q20 Which year groups receive tobacco education within the formal curriculum and where is it taught?

PLEASE TICK ALL THAT APPLY FOR EACH YEAR GROUP IN YOUR SCHOOL

	PSE or Welsh Baccalaureate	Science	Other	Not taught to this year group
Year 7	☐	☐	☐	☐
Year 8	☐	☐	☐	☐
Year 9	☐	☐	☐	☐
Year 10	☐	☐	☐	☐
Year 11	☐	☐	☐	☐
Year 12	☐	☐	☐	☐
Year 13	☐	☐	☐	☐

Q21 Which year groups receive drug education within the formal curriculum and where is it taught?

PLEASE TICK ALL THAT APPLY FOR EACH YEAR GROUP IN YOUR SCHOOL

	PSE or Welsh Baccalaureate	Science	Other	Not taught to this year group
Year 7	☐	☐	☐	☐
Year 8	☐	☐	☐	☐
Year 9	☐	☐	☐	☐
Year 10	☐	☐	☐	☐
Year 11	☐	☐	☐	☐
Year 12	☐	☐	☐	☐
Year 13	☐	☐	☐	☐

Q22 Which year groups receive alcohol education within the formal curriculum and where is it taught?

PLEASE TICK ALL THAT APPLY FOR EACH YEAR GROUP IN YOUR SCHOOL

	PSE or Welsh Baccalaureate	Science	Other	Not taught to this year group
Year 7	☐	☐	☐	☐
Year 8	☐	☐	☐	☐
Year 9	☐	☐	☐	☐
Year 10	☐	☐	☐	☐
Year 11	☐	☐	☐	☐
Year 12	☐	☐	☐	☐
Year 13	☐	☐	☐	☐

Q23 Which year groups receive mental health and wellbeing education (e.g. resilience, emotional literacy) within the formal curriculum and where is it taught?

PLEASE TICK ALL THAT APPLY FOR EACH YEAR GROUP IN YOUR SCHOOL

	PSE or Welsh Baccalaureate	Religious Education	Other	Not taught to this year group
Year 7	☐	☐	☐	☐
Year 8	☐	☐	☐	☐
Year 9	☐	☐	☐	☐
Year 10	☐	☐	☐	☐
Year 11	☐	☐	☐	☐
Year 12	☐	☐	☐	☐
Year 13	☐	☐	☐	☐

Q24 Which year groups receive sex and relationships education within the formal curriculum and where is it taught?

PLEASE TICK ALL THAT APPLY FOR EACH YEAR GROUP IN YOUR SCHOOL

	PSE or Welsh Baccalaureate	Science	Other	Not taught to this year group
Year 7	☐	☐	☐	☐
Year 8	☐	☐	☐	☐
Year 9	☐	☐	☐	☐
Year 10	☐	☐	☐	☐
Year 11	☐	☐	☐	☐
Year 12	☐	☐	☐	☐
Year 13	☐	☐	☐	☐

PERSONAL & SOCIAL EDUCATION (PSE)

Q25a Who is responsible for coordinating PSE provision in the school?
PLEASE TICK ONE BOX ONLY

- ☐ Headteacher
- ☐ Deputy headteacher
- ☐ Assistant headteacher
- ☐ Specialist PSE teacher
- ☐ Other **(PLEASE TICK AND WRITE IN BELOW)**

[✎]

Q25b Which teachers have the **main** responsibility for teaching PSE?
PLEASE TICK ONE BOX ONLY

- ☐ PE teachers
- ☐ Science teachers
- ☐ Specialist PSE / health education teachers
- ☐ RE teachers
- ☐ Form tutors
- ☐ Outside agencies
- ☐ Any classroom teacher / No group has main responsibility
- ☐ Other **(PLEASE TICK AND WRITE IN BELOW)**

[✎]

Q25c If non-specialist PSE / health education teachers teach PSE, is formal training provided to support their delivery of the PSE curriculum?
PLEASE TICK ONE BOX ONLY

- ☐ Yes, compulsory training
- ☐ Yes, optional training
- ☐ No
- ☐ Only specialist teachers teach PSE

Q25d Which of the following do students receive for PSE?
PLEASE TICK ALL THAT APPLY

- ☐ Grades
- ☐ Reports
- ☐ Self-assessment
- ☐ Target setting
- ☐ None of the above

PHYSICAL ACTIVITY & ACTIVE TRANSPORT

Q26a On average, how many days a week can students in years 7 and 10 participate in extra-curricular sport or other structured physical activity (led by staff, volunteers, other students) in the autumn and summer terms, e.g. football club, dance club?

TICK ONE BOX ON EACH ROW FOR AUTUMN TERM AND THEN SUMMER TERM

**Autumn term**	0 days	1-2 days	3-4 days	5 days
Year 7	☐	☐	☐	☐
Year 10	☐	☐	☐	☐

**Summer term**	0 days	1-2 days	3-4 days	5 days
Year 7	☐	☐	☐	☐
Year 10	☐	☐	☐	☐

Q26b Who delivers extra-curricular sports and physical activity in your school?

TICK ALL THAT APPLY

- ☐ PE teachers
- ☐ Other teachers or other school staff
- ☐ Parents
- ☐ Sport Wales Young Ambassadors
- ☐ Other volunteers
- ☐ Externally contracted coaches
- ☐ Local authority or community sports staff, e.g. 5x60 officer
- ☐ School has no extra-curricular sports

Q27 Are the following available to students on site (with or without supervision)?

IF YES, TICK *ALL* TIMES WHEN FACILITY IS AVAILABLE

	No	Yes, as part of PE lessons	Yes, during lunch	Yes, after school
Gymnasium / sports hall	☐	☐	☐	☐
Dance / fitness studio	☐	☐	☐	☐
Swimming pool	☐	☐	☐	☐
Running track	☐	☐	☐	☐
Sports field / grass pitches	☐	☐	☐	☐
Basketball / netball courts	☐	☐	☐	☐
5-a-side football pitches	☐	☐	☐	☐
Playground	☐	☐	☐	☐
Skateboard area	☐	☐	☐	☐
Equipment for team sports	☐	☐	☐	☐

Q28 How many Sport Wales Young Ambassadors does your school currently have?

PLEASE TICK ONE BOX ONLY

- ☐ My school is not in the Sport Wales Young Ambassador scheme
- ☐ 0 (Joined scheme, but yet to recruit Ambassadors)
- ☐ 1
- ☐ 2
- ☐ 3
- ☐ 4
- ☐ 5 or more

Q29 Does your school have a student-led group informing sport and/or physical activity **policies**, e.g. a sport council?
PLEASE TICK ONE BOX ONLY

☐ Yes

☐ No

☐ We are currently developing a group

Q30a Does your school promote active transport in the following ways?
PLEASE TICK ALL THAT APPLY

- Identify and promote safe walking and cycling routes ☐
- Secure covered storage for bicycles and scooters ☐
- Promotion of helmets for cyclists ☐
- Walking promotions, e.g. 'Walk to School Week' ☐
- Cycling proficiency training ☐
- Pedestrian/cyclist entrances ☐
- Collaboration with police/PCSOs to address community/transport safety ☐
- Other **(PLEASE TICK AND WRITE IN BELOW)** ☐

✍ ______________________________

Q30b Does your school:

	Yes	No
Monitor the number of students walking or cycling to school	☐	☐
Set goals to increase the number of students walking or cycling to school	☐	☐

Q31 Does your school have partnerships* with any of the following individuals or groups to help students remain or become physically active?

PLEASE TICK ALL THAT APPLY

- ☐ Families
- ☐ Other schools
- ☐ Local community groups
- ☐ Professional sports clubs
- ☐ Sport Wales or other national sport bodies
- ☐ Private sector businesses or organisations
- ☐ Local authority 5x60 officers
- ☐ Health board
- ☐ Other

* *Partnerships are any formal or informal relationships which exist for a period of time or on an on-going basis and which, in the case of local authorities and health boards, go beyond statutory requirements.*

HEALTHY EATING

Q32 Is the food that your students bring into school ever monitored?

PLEASE TICK ONE BOX ONLY

- ☐ Yes, once a term or more
- ☐ No
- ☐ Yes, less often

Q33 Does your school do any of the following to promote healthy eating?
TICK ALL THAT APPLY

- ☐ Monitor and set goals to increase uptake of free school meals
- ☐ Use a cashless system to support free school meal uptake
- ☐ Clearly identify healthier options in the school canteen
- ☐ Position healthier options at the start of the food service
- ☐ Incentivise healthier options through pricing
- ☐ Incentivise healthier options through other means

Q34a Does your school or any other organisation provide any extra-curricular programmes for learning about food and healthy eating, e.g. cooking clubs? **PLEASE TICK ONE BOX ONLY**

- ☐ Yes, regularly **(GO TO Q34b)**
- ☐ Yes, ad hoc or one off events **(GO TO Q34b)**
- ☐ No **(GO TO Q35a)**

Q34b **IF YES AT Q34a**, are parents invited to take part in these programmes?
PLEASE TICK ONE BOX ONLY

- ☐ Always
- ☐ Rarely
- ☐ Sometimes
- ☐ Never

Q35a Does your school offer a breakfast club before the start of the morning school session? **PLEASE TICK ONE BOX ONLY**

☐ Yes, to all students throughout the year **(GO TO Q35b and c)**

☐ Yes, to some students and/or at certain times of year **(GO TO Q35b and c)**

☐ No **(GO TO Q36)**

Q35b **IF YES AT Q35a,** how many days a week is it offered?
PLEASE TICK ONE BOX ONLY

☐ 1 day

☐ 2 days

☐ 3 days

☐ 4 days

☐ 5 days

Q35c **IF YES AT Q35a,** do students pay for their breakfast at the club?
PLEASE TICK ONE BOX ONLY

☐ Yes, all students taking part pay

☐ Yes, but some students are subsidised on the basis of need

☐ No, it is free of charge

**Q36** **Please tick Yes or No to the following questions about your school dining environment:**	**Yes**	**No**
Does your school's dining room have an adequate number of chairs and tables?	☐	☐
Do students have at least 20 minutes to eat lunch once seated?	☐	☐
Are students who bring packed lunches allowed to sit with those eating school meals?	☐	☐
Does the dining room have freely available drinking water?	☐	☐
Is the dining room always supervised?	☐	☐

If your school has a healthy eating or Food & Fitness policy, please answer question 37. If not, please go to Q38.

Q37a Does your school's healthy eating or Food & Fitness policy cover the types of foods and beverages that can be offered or sold at the following:
PLEASE TICK ONE BOX ON EACH ROW

	Yes	No	Not applicable
In student enterprise projects	☐	☐	☐
At school social events for parents	☐	☐	☐
At school social events for students	☐	☐	☐
For school or student fundraising	☐	☐	☐

Q37b Does your school's healthy eating or Food & Fitness policy include guidance or requirements on the types of foods and beverages that students can bring into school, e.g. in packed lunches?
PLEASE TICK ONE BOX ONLY

☐ Yes, guidance **(GO TO Q37c)**
☐ No **(GO TO Q38)**
☐ Yes, requirements **(GO TO Q37c)**

Q37c **IF YES AT Q37b**, does this include food taken on school trips?
PLEASE TICK ONE BOX ONLY

☐ Yes
☐ No

Q38 Does your school have a student-led group informing nutrition <u>**policies**</u>, e.g. a school nutrition action group (SNAG)?
PLEASE TICK ONE BOX ONLY

☐ Yes
☐ No
☐ We are currently developing a group

TOBACCO, DRUGS & ALCOHOL

Q39a Has your school used the following in the last two years:

PLEASE TICK ONE BOX ON EACH ROW

	Yes	No
Resources from the All Wales School Liaison Core Programme for its alcohol or drug teaching?	☐	☐
No Smoking Day resources for its tobacco teaching?	☐	☐
Theatre in education for its tobacco, alcohol or drug teaching?	☐	☐

		Yes	No
**Q39b**	Has your school participated in the ASSIST smoking prevention programme delivered by Public Health Wales?	☐	☐
**Q39c**	Has your school participated in the 'JustB' smoking prevention programme delivered by Public Health Wales?	☐	☐

Q40a Does your school offer any type of tobacco cessation support to students?

PLEASE TICK ONE BOX ONLY

☐ Yes **(GO TO Q40b)**

☐ No **(GO TO Q41)**

Q40b **IF YES AT Q40a,** are these offered:

PLEASE TICK ALL THAT APPLY

☐ At school

☐ Through referral to an off-site service or initiative

Q41 Does your school have a specified pathway or a referral process in place to provide expertise and resources for students who:

PLEASE TICK ONE BOX ON EACH ROW

	Yes	No
Misuse drugs	☐	☐
Misuse alcohol	☐	☐

If your school has a smoking and tobacco use policy, please answer question 42. If not, please got to Q43.

Q42a Does your school's smoking and tobacco use policy prohibit tobacco use in the following locations?

PLEASE TICK ONE BOX ON EACH ROW

	Yes	No
School grounds during school hours	☐	☐
School grounds outside of school hours	☐	☐
Private vehicles on school grounds	☐	☐
School events off school grounds	☐	☐

Q42b Does the policy apply to everyone, including students, staff, families and visitors? **PLEASE TICK ONE BOX ONLY**

☐ Yes

☐ No

Q42c Does your school prohibit *possession* of tobacco products for students on school property as well as *use*? **PLEASE TICK ONE BOX ONLY**

☐ Yes

☐ No

Q42d Does the policy cover the use of electronic cigarettes, also called personal vaporizers or electronic nicotine delivery systems (ENDS)? **PLEASE TICK ONE BOX ONLY**

☐ Yes

☐ No

MENTAL HEALTH & WELLBEING

Q43 Is cyber-bullying included in your policies on bullying?

PLEASE TICK ONE BOX ONLY

- ☐ Yes
- ☐ No

Q44 Does your mental health and wellbeing policy include staff mental health?

PLEASE TICK ONE BOX ONLY

- ☐ Yes
- ☐ No
- ☐ No mental health and wellbeing policy

Q45 Does your suicide prevention and/or post-suicide care policy include self-harm?

PLEASE TICK ONE BOX ONLY

- ☐ Yes
- ☐ No
- ☐ No suicide prevention and/or post-suicide care policy

Q46 Does your school use any of the following:

TICK ALL THAT APPLY

- ☐ Social and Emotional Aspects of Learning (SEAL)
- ☐ The Student Assistance Programme (SAP)
- ☐ Emotional Literacy Support Assistants (ELSA)
- ☐ None of the above

SEX & RELATIONSHIPS

Q47 Who has the main responsibility for teaching sex and relationships education (SRE)?
PLEASE TICK ONE BOX ONLY

- ☐ Science teachers
- ☐ Specialist SRE / health education teachers
- ☐ RE teachers
- ☐ Form tutors
- ☐ School nurse
- ☐ Any classroom teacher / no group has responsibility
- ☐ Outside agencies
- ☐ Other (**PLEASE TICK AND WRITE IN BELOW**)

✍ ____________________

Q48 What proportion of parents remove their child/ren from SRE?
PLEASE TICK ONE BOX ONLY

- ☐ Less than 5%
- ☐ 5-10%
- ☐ 11-20%
- ☐ 21-50%
- ☐ More than 50%

Q49 Does your school currently provide any staff training in safeguarding young people **specifically about issues relating to sexual health and relationships**?
PLEASE TICK ONE BOX ONLY

- ☐ Yes
- ☐ No

Q50a Does your school have an on-site 'drop-in' service specifically for sexual health advice?

PLEASE TICK ONE BOX ONLY

☐ Yes **(GO TO Q50b)**

☐ No **(GO TO Q51)**

Q50b **IF YES AT Q50a**, who is the service provided by?

PLEASE TICK ALL THAT APPLY

☐ School nurse

☐ Local authority youth workers

☐ NHS health visitor

☐ Charity youth workers

☐ Other **(PLEASE TICK AND WRITE IN BELOW)**

✍

Q51 Does your school have on-site provision of free condoms for school students (including distribution using the C-Card scheme)?

PLEASE TICK ONE BOX ONLY

☐ Yes

☐ No

VIOLENCE AGAINST WOMEN AND GIRLS (GENDER BASED VIOLENCE)

Q52 Did staff at your school receive any training on the following issues in the previous two academic years (2013/14 and 2014/15)?

PLEASE TICK ONE BOX ON EACH ROW

	No	Yes, some staff	Yes, all staff
Understanding what violence against women, domestic abuse and sexual violence is	☐	☐	☐
Recognising the signs of violence against girls aged 11-16	☐	☐	☐
How to take appropriate action where signs of violence against girls aged 11-16 are observed	☐	☐	☐

HEALTH SERVICE PROVISION

Q53a Does your school have an NHS School Nurse?

PLEASE TICK ONE BOX ONLY

- ☐ Yes **(GO TO Q53b and c)**
- ☐ No **(GO TO Q54a)**

Q53b **IF YES AT Q53a**, How often is the nurse service on-site and available to students?

- ☐ Every school day
- ☐ 3-4 times per week
- ☐ 1-2 times per week
- ☐ Once a fortnight
- ☐ Less often
- ☐ Nurse visits on request only

Q53c **IF YES AT Q53a**, Which of the following does the NHS school nurse do at your school?

TICK ALL THAT APPLY

- ☐ Supports the school in managing attendance
- ☐ One-to-one student advice and support on an ad hoc basis
- ☐ A regular drop-in service for students
- ☐ Advice on managing health issues in the school setting for individual pupils
- ☐ Smoking cessation support
- ☐ Safeguarding
- ☐ Contributes to teaching on health and wellbeing topics in the formal curriculum
- ☐ None of the above

Q54a Is there a school-based counselling service?
PLEASE TICK ONE BOX ONLY

☐ Yes **(GO TO Q54b)**

☐ No **(GO TO Q55a)**

Q54b **IF YES AT Q54a,** how often is the service available?
PLEASE TICK ONE BOX ONLY

☐ Every school day
☐ 2 – 4 times a week
☐ Once a week
☐ Less often
☐ On request only

Q55a Is there a school-based educational psychologist available to students?
PLEASE TICK ONE BOX ONLY

☐ Yes **(GO TO Q55b)**

☐ No **(GO TO Q56a)**

Q55b **IF YES AT Q55a,** how often is the psychologist available?
PLEASE TICK ONE BOX ONLY

☐ Every school day
☐ 2 – 4 times a week
☐ Once a week
☐ Less often
☐ On request only

Q56a Are students informed of other local, confidential counselling services they can use?

PLEASE TICK ONE BOX ONLY

☐ Yes **(GO TO Q56b)**

☐ No **(GO TO Q57)**

Q56b **IF YES AT Q56a,** can students attend such services during school hours?

PLEASE TICK ONE BOX ONLY

☐ Yes

☐ No

☐ Only in exceptional circumstances

BEHAVIOUR & DISCIPLINE

Q57 Does your school use isolation to manage student behaviour?

PLEASE TICK ONE BOX ONLY

☐ Yes ☐ No

Q58 How many exclusions were there at your school in the 2014/15 school year?

PLEASE INDICATE THE TOTAL NUMBER OF *EXCLUSIONS* OF EACH TYPE LISTED, NOT THE NUMBER OF STUDENTS

Fixed term exclusions of 5 days or less ☐☐

Fixed term exclusions of over 5 days ☐☐

Permanent exclusions ☐☐

Q59 Does your school currently use the following restorative practices in your approach to student discipline?

PLEASE TICK ALL BOXES THAT APPLY

- ☐ Circle time
- ☐ Restorative conference
- ☐ Peer mediation
- ☐ Other restorative practices

Q60 Does your school work with community police officers on restorative practices?

PLEASE TICK ONE BOX ONLY

☐ Yes ☐ No

The aim of this section of the questionnaire is to scope schools' existing practices around self-harm.

The information you provide will inform the development of interventions that address self-harm and are appropriate and feasible in the school setting.

Health priorities and interventions in your school

Q61 The following are a list of health related areas often dealt with in schools through teaching and other activities. What level of importance is given to each by your school?

Please note, this may not reflect your personal view.

PLEASE RANK THE 9 HEALTH AREAS IN ORDER OF IMPORTANCE, STARTING WITH [1] AS THE MOST IMPORTANT TO YOUR SCHOOL.

	Rank number
Sex and relationships	☐
Suicide prevention	☐
Smoking	☐
Emotional health and wellbeing	☐
Alcohol	☐
Healthy eating	☐
Self-harm	☐
Physical activity	☐
Drugs	☐

Health priorities and interventions in your school

Q62 The following are a list of different types of interventions that schools may use to address a range of health related topics. What is the level of usefulness (for both staff and students) of these intervention types in addressing the health areas prioritised by your school?

PLEASE TICK ONE BOX ON EACH ROW

	Very high	High	Average	Low	Very low
Posters and leaflets	☐	☐	☐	☐	☐
One-to-one intervention	☐	☐	☐	☐	☐
Targeted group support	☐	☐	☐	☐	☐
Student peer support	☐	☐	☐	☐	☐
Curriculum lessons	☐	☐	☐	☐	☐
Staff information and training	☐	☐	☐	☐	☐
External agency intervention	☐	☐	☐	☐	☐
Whole school approaches (e.g. addressing school policies and relationships)	☐	☐	☐	☐	☐
Other **(PLEASE WRITE IN BELOW)**	☐	☐	☐	☐	☐

✍

Self-harm, your students and school

Q63 Approximately 10% of young people in the UK self-harm, so for the purpose of this survey we define an **'average' level of self-harm within a school** as **10% of students** intentionally harming themselves.

How do you think the level of self-harm amongst students in your school compares to the average?

For each type of self-harm behaviour listed below, please indicate whether you think the proportion of your student body that engage in the behaviour is very high, high, average (~10%), low or very low.

PLEASE TICK ONE BOX ON EACH ROW

	Very high	High	Average (~ 10%)	Low	Very low
Cutting	☐	☐	☐	☐	☐
Poisoning	☐	☐	☐	☐	☐
Over-eating or under-eating	☐	☐	☐	☐	☐
Burning of the skin	☐	☐	☐	☐	☐
Hitting or scratching self	☐	☐	☐	☐	☐
Excessive exercise	☐	☐	☐	☐	☐
Hair pulling	☐	☐	☐	☐	☐
Excessive alcohol or drug use	☐	☐	☐	☐	☐
Other **(PLEASE WRITE IN BELOW)**	☐	☐	☐	☐	☐

✍ ______________________________

Self-harm prevention and activities

For the purposes of this survey we define self-harm as any behaviour that is intended to intentionally hurt oneself. It may or may not be associated with suicidal intent.

Self-harm prevention and activities

Q64 Which of the following self-harm prevention and intervention activities are delivered in your school?

For each item, please select 'Yes. Routine provision' *if it is provided in your school at least on an annual basis. If an item is provided on an ad-hoc, one-off basis, please tick* 'Yes. One-off provision'. *If an item is not provided and you do not think it needs to be, please tick* 'No'. *If an item is not provided but you would like it to be, please tick* 'No, but would like to'.

		Yes. Routine provision	Yes. One-off provision	No	No, but would like to
A	Assemblies themed around self-harm	☐	☐	☐	☐
B	PSE sessions themed around self-harm	☐	☐	☐	☐
C	An on-site counsellor (paid or voluntary)	☐	☐	☐	☐
D	A drop-in health service, provided by school nurse or other health professional	☐	☐	☐	☐
E	Specialist self-harm prevention training for students	☐	☐	☐	☐
F	Posters on display about self-harm	☐	☐	☐	☐
G	Visits from outside speakers or organisations to talk to students about self-harm	☐	☐	☐	☐
H	Clear procedures known to all staff for identifying and supporting students who self harm	☐	☐	☐	☐
I	Training for teachers and staff about self-harm	☐	☐	☐	☐
J	Regular contact with relevant health services, e.g. Child & Adolescent Mental Health Service (CAMHS)	☐	☐	☐	☐

Please list below any other prevention and intervention activities undertaken by your school

K

L

M

N

O

Self-harm prevention and activities

Q65 Considering the prevention and intervention activities listed in Q64 (options A to O), which **five** do you consider to be **most useful** for a school to provide?

PLEASE ENTER '1' BESIDE THE ACTIVITY YOU CONSIDER MOST USEFUL THROUGH TO '5' NEXT TO THE FIFTH MOST USEFUL

- A ☐
- B ☐
- C ☐
- D ☐
- E ☐
- F ☐
- G ☐
- H ☐
- I ☐
- J ☐
- K ☐
- L ☐
- M ☐
- N ☐
- O ☐

Self-harm prevention and activities

Q66 If provided, who contributes to self-harm prevention or intervention activities in your school?

PLEASE TICK ONE BOX ON EACH ROW

	Yes	No
Teachers	☐	☐
Teaching support staff	☐	☐
Pastoral care team	☐	☐
School senior management	☐	☐
Students	☐	☐
School nurse	☐	☐
School counsellor	☐	☐
Mental health specialists (e.g. CAMHS)	☐	☐
Other health professional **(PLEASE TICK AND WRITE IN BELOW)**	☐	☐
✍		
Voluntary sector worker, e.g. Samaritan volunteer **(PLEASE TICK AND WRITE IN BELOW)**	☐	☐
✍		
Other, e.g. youth worker **(PLEASE TICK AND WRITE IN BELOW)**	☐	☐
✍		

Self-harm prevention and activities

Q67a Have school staff received training in self-harm prevention and intervention?

TICK ONE BOX ONLY

- ☐ Yes, mandatory training **(GO TO Q67b)**
- ☐ Yes, voluntary training **(GO TO Q67b)**
- ☐ No **(GO TO Q68a)**
- ☐ Don't know **(GO TO Q68a)**

Q67b **IF YES AT Q67a,** please state:

Training provider	
Training funder	

Future provision in self-harm prevention and intervention

Q68a How would you rate the adequacy of lessons, activities and services that address self-harm in your school?

TICK ONE BOX ONLY

- ☐ Very low
- ☐ Low
- ☐ Moderate
- ☐ High
- ☐ Very high

Q68b Please explain your reasons for selecting this level.

Future provision in self-harm prevention and intervention

Q69 How much of a barrier are the following to delivering self-harm prevention and intervention activities in your school?

PLEASE TICK ONE BOX ON EACH ROW

	Major barrier	Minor barrier	Not a barrier
Self-harm is not seen as a problem by senior management in my school	☐	☐	☐
Self-harm is not seen as a problem by teachers in my school	☐	☐	☐
Other health topics are given higher priority in health related lessons and activities	☐	☐	☐
A lack of staff time to deliver self-harm related activities	☐	☐	☐
School staff are not adequately trained in self-harm to be able to deliver activities	☐	☐	☐
Fear about encouraging self-harm in students	☐	☐	☐
A lack of available resources such as worksheets, videos and ideas for activities	☐	☐	☐
Pressures to deliver core curriculum subjects mean teachers have little time left to spend on health related activities	☐	☐	☐
School is not an appropriate place to deal with this topic	☐	☐	☐
Students fail to engage with activities on this topic	☐	☐	☐
Other **(PLEASE TICK AND WRITE IN BELOW)**	☐	☐	☐

✍

Future provision in self-harm prevention and intervention

Q70 Would your school be prepared to participate in future research to develop student self-harm prevention and intervention activities for delivery in schools?

TICK ONE BOX ONLY

- ☐ Yes
- ☐ No
- ☐ Don't know

Thank you for completing the questionnaire.

Please return your questionnaire to the School Health Research Network in the freepost envelope provided.
